# Death cause and age at death among individuals with diabetes: a 40-year retrospective Brazilian study

**DOI:** 10.20945/2359-4292-2026-0034

**Published:** 2026-04-01

**Authors:** Lucas Casagrande Passoni Lopes, Marina dos Santos de Carvalho Pinto, Mauro Wieczorek, Carlos Antonio Negrato

**Affiliations:** 1 Universidade de São Paulo, Faculdade de Medicina de Bauru, Bauru, SP, Brasil

**Keywords:** Diabetes, mortality, death causes, sex, diabetes type

## Abstract

**Objective:**

To evaluate the association between specific/all-cause mortality and the individual’s age at death.

**Materials and methods:**

This was a retrospective study that employed STROBE guidelines to its development. Medical records of 1,367 individuals diagnosed with diabetes mellitus (DM) who died between 1981 and 2021, in Bauru, São Paulo state, Brazil, were evaluated. Demographic, clinical, and obituary data were collected from medical records and death certificates, serving as adjustment variables in the analysis. Statistics were developed by using Cox-regression models.

**Results:**

The final model, adjusted by male sex and T2DM, revealed Hazard Ratios of 0.74 (95% CI: 0.59-0.92; p < 0.01) for infectious causes and 1.92 (95% CI: 1.12-3.37; p < 0.01) for gastrointestinal conditions. These findings suggest that infectious causes were associated with later ages at death, while gastrointestinal conditions were related to younger ages at death. The other evaluated variables and groups of death causes did not show statistical significance.

**Conclusion:**

Our study demonstrates that sex and DM type significantly influenced age at death. These findings emphasize the importance of considering demographic and clinical factors when assessing mortality patterns in DM, contributing to improved risk stratification and clinical management strategies.

## INTRODUCTION

Diabetes mellitus (DM) encompasses a group of metabolic disorders whose hallmark is hyperglycemia ^(1)^. The most common types of DM are type 1 DM (T1DM), which derives, predominantly, from an autoimmune condition; and type 2 DM (T2DM), whose most meaningful characteristic is insulin resistance ^(1)^.

Globally, it is estimated that more than 500 million individuals are living with DM in 2025 ^(2)^. These estimates are expected to rise to over 1.31 billion by 2050, mostly due to the increasing prevalence of T2DM ^(2)^. In Brazil, DM affects almost 10% of the population, with significant regional disparities and high underreporting rates ^(2)^. The rising prevalence of DM in Brazil mirrors global trends, underscoring the urgent need for robust public health interventions to address its growing burden ^(2)^.

Regarding mortality, DM is a leading contributor to death, due to its systemic nature its systemic nature ^(3)^. Indeed, a long-standing hyperglycemia, through complex pathophysiological mechanisms, causes protein glycation and cellular dysfunction, disrupting the individual’s homeostasis ^(4)^. This process generates endothelial dysfunction that contributes to significant occurrence of cardiovascular (CVD) events and functional impairment of the kidneys in individuals with DM ^(4)^. Thereto, individuals with DM tend to present higher mortality rates when compared with the whole population ^(3)^.

Under this perspective, several studies have been conducted comparing mortality patterns between patients with and without DM, particularly concerning specific and all-cause mortality ^(3-5)^. In parallel, other studies have focused exclusively on mortality risk due to distinct causes among patients with DM, using age as the crucial adjustment factor in their analyses ^(3-5)^. However, a significant gap exists in understanding the interplay between age at death and its association with specific and all-cause mortality in patients with DM ^(5)^. Therefore, the present study aimed to evaluate the association between specific/all-cause mortality and the age at death.

## MATERIALS AND METHODS

### Study design

This was a retrospective study conducted following the STROBE guidelines.

**Figure 1:**
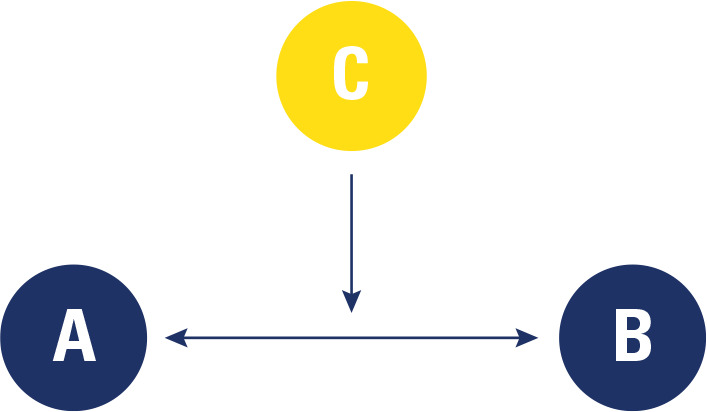
Study design and aims graphic representation. (**A**) Patients’ age at death; (**B**) Patients’ death cause; (**C**) Other considered variables such as sex, ethnicity, marital status, death’s decade, DM type, DM duration, proposed treatment, BMI.

The main objective of this study was to evaluate the association between specific/all-cause mortality and the age at death. Since other variables such as sex, ethnicity, marital status, decade of death, DM type, DM duration, treatment modality and body mass index (BMI) may influence this relationship, they were also considered in the final analysis as adjustment variables. Consequently, the secondary aim of this study was to evaluate the interaction between these variables on patients specific and all-cause mortality on the age of death. **Figure 1** shows a graphic representation of the study design and aims.

### Setting

The study was carried out in Bauru city, located in the central-western region of São Paulo state, Brazil, with data collection being carried out between 1981 and 2021.

Previous studies conducted in Bauru with specific populations pointed to an increasing trend in DM cases, both for T1DM and for T2DM. However, precise epidemiological data on these conditions remain scarce and limited ^(6)^.

All patients were treated and followed at the same endocrinology clinic, which simultaneously acts as a private practice and as a referral center for the public health system in Bauru and surrounding areas. This dual function ensures a balanced representation of the local population, as approximately half of the patients were privately insured or self-paying, and the other half were referred by the public sector - specifically, the *Associação dos Diabéticos de Bauru* (ADB).

ADB is a public, non-profit organization affiliated with the *Faculdade de Medicina de Bauru, Universidade de São Paulo* (FMBRU-USP). It provides free care to individuals with DM and works in close partnership with the mentioned endocrinology clinic.

While care in both settings was coordinated by the same endocrinologist throughout the 40-year study period, ADB also includes a multidisciplinary team with nurses and other health professionals involved in patients’ education and support, as well as an important work driven by medical students.

Importantly, all provided care was guided by a personalized and humanized approach. There was no rigid schedule for follow-up visits; instead, the interval between consultations was individually determined based on the patients’ clinical needs, personal circumstances, and availability. This approach aligns with the concept of a therapeutic care plan, aiming to respect the uniqueness of each patient’s health journey.

### Participants

This study included 1,367 individuals with a confirmed diagnosis of DM who died between 1981 and 2021. Patients with a documented diagnosis of DM and complete data in both medical records and death certificates were included. Patients who did not meet these criteria were excluded from the analysis.

### Variables

The primary outcome was individuals’ age at death. Explanatory variables included: Demographic: sex, ethnicity (self-reported: White, Black, Brown, Yellow, Indigenous), marital status (single, married, widowed, divorced), and decade of death; Clinical: DM type (T1DM or T2DM), DM duration (in years), treatment modality (diet, oral antidiabetic agents, insulin, or combinations), and BMI; Mortality: cause of death.

Causes of death were grouped into six main categories: CVD, external causes (E), acute complications of chronic diseases (AC), infectious causes (I), neoplasms (N), gastrointestinal diseases (GD), and Ill-defined causes (ID). In the present study, causes of death were reclassified into broader etiological categories based on the underlying cause of death, rather than the immediate or terminal mechanisms reported in death certificates. Conditions such as cardiorespiratory arrest, multiple organ failure, shock, and respiratory failure were considered final common pathways of death and were therefore grouped according to their most plausible underlying etiological cause, following epidemiological, WHO, and Brazilian Health Minister recommendations for mortality analysis. This reclassification aimed to improve analytical coherence, reduce misclassification bias, and allow meaningful comparisons across cause-specific mortality groups.

The study also examined interactions between the independent variables and their influence on the age at death.

### Data sources and measurement

Demographic and clinical data were extracted from patients’ medical records, maintained by the same endocrinologist to ensure consistency. Obituary data were manually collected from two official Registry Offices in Bauru, with oversight by authorized professionals. Death certificates followed Brazil’s standardized format, prioritizing the underlying cause of death in epidemiological analyses.

BMI was calculated dividing weight (kg) by the square of the height (m^2)^ and categorized into: underweight (<18.5), normal weight (18.6-24.9), overweight (25-29.9), obesity grade I (30-34.9), grade II (35-39.9), and grade III (>40) ^(6)^. Diagnosis and treatment of DM were done according to Brazilian Diabetes Society and American Diabetes Association guidelines ^(7,8)^.

### Bias

To minimize bias, all clinical data were collected by a single physician using uniform documentation practices. However, retrospective data collection from death certificates may be limited due to inconsistencies in how causes of death were recorded, especially in Ill-defined cases.

### Study size

The initial dataset included 1,367 individuals. The final analytical sample resulted from the availability of valid and complete data records. No sample size calculation was performed due to the retrospective design of the study and full inclusion of eligible records.

### Quantitative variables

Quantitative variables such as DM duration, decade of death, and BMI were analyzed both as continuous and categorical variables. Ethnicity was recategorized as White vs. non-White to enhance statistical power.

### Statistical methods

Normality of continuous variables was assessed using the Shapiro-Wilk test. Descriptive statistics were followed by bivariate analyses using Chi-square, Student’s t-test, and ANOVA to examine associations between demographic/clinical variables and age at death and cause of death.

Variables with statistically significant associations (p < 0.05) were included in survival analysis using Kaplan-Meier curves and Cox proportional hazards models, which death was the event of interest. The likelihood ratio test was used to compare nested models. Hazard ratios (HR) > 1 indicated higher risk of earlier death; HR < 1 indicated longer survival. In this context, Cox proportional hazards models were applied to assess the temporal ordering of age at death across cause-of-death groups rather than its classical approach.

The average age at death across the cohort served as the reference for comparison. All statistical analyses were performed using R software version 4.2.0, and graphs were generated using R^®^ and Canva^®^.

### Ethics approval

This study was approved by the Research Ethics Committee of the School of Dentistry of the University of São Paulo (Protocol No. 37022220.0.0000.5417). Judicial authorization was obtained for accessing and analyzing death certificates.

## RESULTS

A total of 555 individuals were excluded from the analysis due to inconsistencies identified during the data selection process. Among them, 208 were confirmed not to have been residents of Bauru at the time of death, which made it impossible to retrieve their obituary data from other local registry offices. Furthermore, 247 patients lacked reliable information regarding the year of death or had no identifiable death certificate available in the municipal records. These issues were primarily attributed to duplicated names that prevented proper identification, absence of key identifiers such as maternal names, or misspellings and inconsistencies on how names were recorded in official documents. While the exact reasons for each missing record could not be fully verified, these hypotheses were suggested by registry office staff as the most likely explanations. Therefore, the final sample was composed of 812 individuals.

The mean age at death among patients was 72.35 ± 14.06 years. **Supplementary table 1** provides an overview of the mean ages at death and their corresponding confidence intervals across all evaluated subgroups.

Statistical analysis revealed a significant association between patients’ age at death and cause of death with sex (p < 0.01) and DM type (p < 0.01). Other variables did not show statistical significance (**Supplementary table 1**).

An initial survival model, excluding interactions between sex and DM type, was created. Subsequently, separate models were developed for each subgroup: men, women, T1DM, and T2DM. Statistically significant findings were observed only in the models for men (p = 0.007) and T2DM (p = 0.006). Based on these findings, a final model was developed, including both men and T2DM concurrently as shown in **Table 1**.

**Table 1:** Cox regression results

Models	CVD	AC	E	I	N	ID	GD	Model’s p-value
Without subgroups	1.09; 0.97 - 1.22; 0.14	0,91; 0.71 - 1.16; 0.45	0.94; 0.55 - 1.60; 0.84	0.80; 0.69 - 0.93; < 0.01	1.18; 0.93 - 1.49; 0.15	1.09; 0.90 - 1.30; 0.38	1.50; 0.98 - 2.29; 0.06	0.003
Men	1.10; 0.92 - 1.32; 0.25	0.82; 0.53 - 1.28; 0.38	0.88; 0.48 - 1.62; 0.70	0.74; 0.60 - 0.91; < 0.01	1.14; 0.83 - 1.56; 0.42	1.25; 0.96 - 1.63; 0.09	1.97; 1.15 - 3.35; 0.01	0.002
Women	1.08; 0.92 - 1.27; 0.30	1.01; 0.75 - 1.36; 0.93	0.94; 0.30 - 2.95; 0.91	0.85; 0.70 - 1.03; 0.10	1.16; 0.82 - 1.64; 0.89	0.98; 0.77 - 1.26; 0.38	1.03; 0.51 - 2.01; 0.91	0.60
T1DM	1.03; 0.57 - 1.89; 0.92	1.03; 0.31 - 3.36; 0.96	0.52; 0.16 - 1.70; 0.27	0.74; 0.35 - 1.51; 0.40	^*^	1.72; 0.91 - 3.23; 0.09	1.07; 0.14 - 7.90; 0.94	0.45
T2DM	1.09; 0.98 - 1.28; 0.13	0.92; 0.71 - 1.17; 0.47	1.19; 0.66 - 2.15; 0.57	0.81; 0.69 - 0.93; < 0.01	1.22; 0.97 - 1.55; 0.08	1.22; 0.97 - 1.55; 0.88	0.49; 0.97 - 2.30; 0.06	0.004
Men and T2DM	1.12; 0.93 - 1.34; 0.21	0.79; 0.51 - 1.25; 0.32	1.10; 0.55 - 2.22; 0.78	0.74; 0.59 - 0.92; < 0.01	1.17; 0.85 - 1.60; 0.34	1.17; 0.88 - 1.54; 0.26	1.93; 1.12 - 3.37; 0.01	0.004

Presentation standard: T1DM, type 1 diabetes; T2DM, type 2 diabetes; Hazard Ratio; 95% Confidence Interval, p-value.

* , an insufficient sample to allow statistical analysis.

CVD: cardiovascular diseases; E: external causes; AC: acute complications of chronic diseases; I: infectious causes; N: neoplasms; GD: gastrointestinal diseases; ID: Ill-defined causes.

p: p-value; CVD: cardiovascular diseases; E: external causes; AC: acute complications of chronic diseases; I: infectious causes; N: neoplasms; GD: gastrointestinal diseases; ID: Ill-defined causes.

Patients who died from infectious causes had a notably reduced likelihood of the event of interest occurring (death at a specific age) when compared to the reference group in the models that demonstrated statistical significance. Specifically, the likelihood of the interest event occur was reduced by approximately: 20% (HR: 0.80; 95% CI: 0.69-0.93; p < 0.01) in the model including only causes of death; 26% (HR: 0.74; 95% CI: 0.60-0.91; p < 0.01) in the model focusing men; and 19% (HR: 0.81; 95% CI: 0.69-0.93; p < 0.01) in the model focusing T2DM. This suggests that patients who died from infectious causes tended to present higher age at death **Table 1**.

Interestingly, patients who died from gastrointestinal causes showed a significantly higher likelihood of the interest event occurring than the reference group in the model that considered men as an adjustment variable (HR: 1.97; 95% CI: 1.15-3.35; p < 0.01). This suggests that patients who died from gastrointestinal causes tended to present lower ages at death. Other variables did not exhibit statistical significance in any model (**Supplementary table 1**).

**Figure 2:**
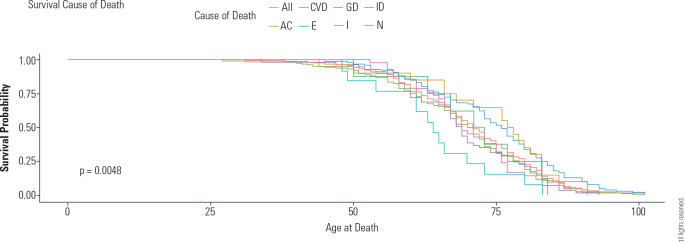
Kaplan-Meier curve for the final model.

**Supplementary figure 1:**
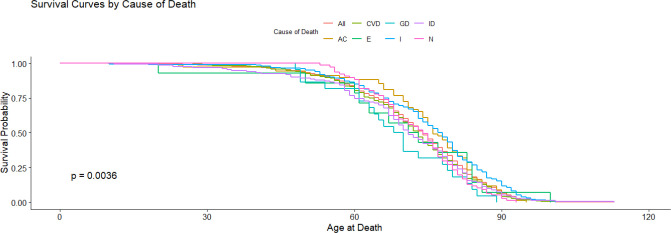
Kaplan-Mayer curves without adjustment variables.

**Supplementary figure 2:**
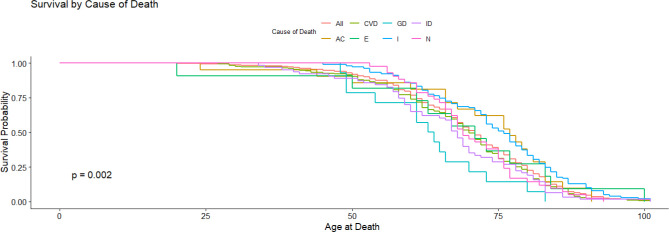
Kaplan-Mayer curves adjusted by men’s model.

**Supplementary figure 3:**
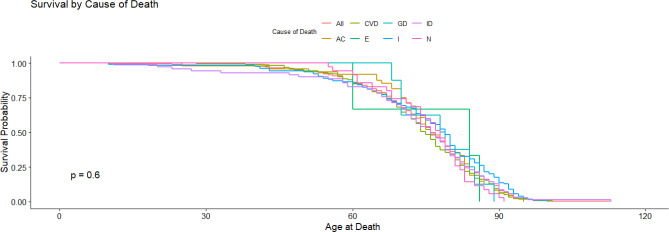
Kaplan-Mayer curves adjusted by women’s model.

**Supplementary figure 4:**
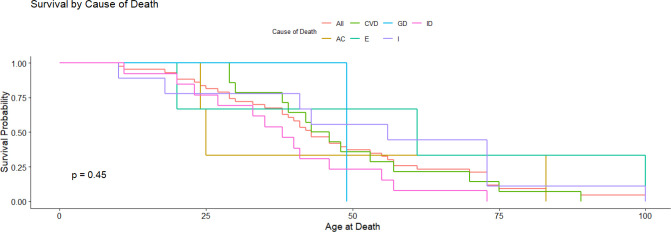
Kaplan-Mayer curves adjusted by T1DM’s model.

**Supplementary figure 5:**
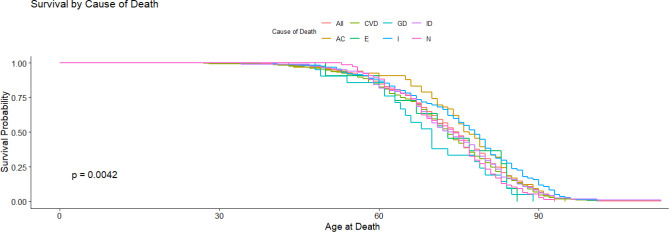
Kaplan-Mayer curves adjusted by T2DM’s model.

In the final model, patients who died from infectious causes had a likelihood of the interest event occurring of approximately 26% lower than the reference group (HR: 0.74; 95% CI: 0.59-0.92; p < 0.01).; and patients who died from gastrointestinal causes exhibited a 93% higher likelihood of the interest event occurring compared to the reference group (HR: 1.92; 95% CI: 1.12-3.37; p < 0.01) - **Figure 2**. These results align with the trends displayed by the other models. **Supplementary figures 1 to 5** bring the other models’ graphical presentations.

Presentation standard: Hazard Ration; 95% Confidence Interval; p-value. Cardiovascular diseases (CVD), external causes (E), acute complications of chronic diseases (AC), infectious causes (I), neoplasms (N), gastrointestinal diseases (GD), and Ill-defined causes (ID).

## DISCUSSION

It was found that patients who died from infectious causes tended to be older at death, while those who died due to gastrointestinal tract-related causes tended to die at younger ages. The other causes of death did not present statistical significance. Some conditions such as male sex and T2DM, seem to modulate the association between age at death and its causes. The other conditions did not show statistical significance.

In the present study, it was found that patients who died from infectious causes tended to be older at the time of death. Studies from the Netherlands have shown that older adults with DM tend to be more frequently hospitalized and decease from infectious conditions compared with younger individuals with DM ^(9,10)^. Possibly, these findings are explained by an interplay between immunosenescence and the chronic immune dysfunction associated with it, which makes older individuals with DM more susceptible not only to acquiring infections, but also to dying from them ^(9,10)^. However a Taiwanese study suggested that the association between DM, infectious diseases and mortality may be mediated by glycemic control levels and the presence of individuals’ comorbidities, proposing a more complex relationship between these variables ^(11)^. Under this perspective, differences in healthcare systems, early detection, and effective treatment protocols may explain these conflicting observations ^(11)^.

Interestingly, our study observed a significant mortality burden attributed to gastrointestinal tract-related diseases. These findings are consistent with the results of an Italian study, which found that young patients with DM tended to die more from gastrointestinal causes compared to other conditions ^(12)^. However, a British study observed that the burden of gastrointestinal causes of death was higher in older individuals with DM ^(13)^. These discrepancies may be attributed to regional differences in cause-of-death certification, access to early interventions, or the possibility that undiagnosed or misclassified complications (e.g., acute pancreatitis, severe hepatic steatosis) were more prevalent in specific study cohorts ^(12,13)^.

Our study identified a high proportion of deaths attributed to gastrointestinal causes among individuals with DM. This finding aligns with a North American cohort study that also reported a substantial burden of gastrointestinal mortality in this population ^(14)^. This may be explained by the high prevalence of non-alcoholic fatty liver disease (NAFLD), steatohepatitis, and other chronic gastrointestinal complications commonly associated with T2DM, especially when coexisting with obesity and metabolic syndrome ^(14)^. These conditions often progress silently and may only be recognized in advanced stages, contributing to increased mortality ^(14)^. Conversely, a British cohort study reported a low proportion of deaths attributed to gastrointestinal causes among individuals with DM ^(15)^. This discrepancy may reflect underdiagnosis and underreporting of these conditions, especially when death certificates prioritize more apparent causes such as cardiovascular events ^(15)^. Moreover, the predominance of macrovascular complications as the leading cause of death in individuals with DM - particularly in high-income countries - may overshadow the contribution of gastrointestinal diseases to overall mortality ^(14,15)^.

In our analysis, cardiovascular causes of death did not show a statistically significant association with age at death. This is consistent with findings from a North American population-based study that also failed to observe a strong link between specific causes of death such as Alzheimer and the age at which individuals died ^(16)^. One possible explanation is that people with DM frequently live with multiple comorbidities ^(16)^. As a result, the impact of any single condition - such as CVD - on age at death may be diluted within a broader pattern of complex, overlapping health issues. On the other hand, multicenter cohort studies have demonstrated important age-related patterns in the causes of death among patients with DM. For instance, a large international meta-analysis showed that cardiovascular risk is higher among those with early-onset or long-standing DM, contributing to younger age at death due to cardiac and cerebrovascular events ^(17)^. In parallel, neoplastic causes tend to be more commonly found among older individuals with DM, particularly liver, pancreatic, and colorectal cancers ^(18)^. These findings are likely driven by the cumulative effects of chronic hyperglycemia, insulin resistance, and systemic inflammation, which increase cancer risk over time ^(18)^. Thus, while DM may accelerate the development of certain neoplasms, their clinical impact tend to become more pronounced in later life ^(17,18)^.

We also observed that male sex significantly modified the association between age and cause of death. This was also supported by a Canadian study that found a significant interaction between sex and cause-specific mortality patterns, with men showing stronger age-related associations than women ^(19)^. The authors attributed these differences to poorer glycemic control and higher rates of macrovascular complications and infections among men, all of which contribute to earlier mortality ^(19)^. Interestingly, the same study highlighted that women with T2DM had proportionally higher cardiovascular mortality compared to men, especially when compared to women without DM ^(20)^. This may reflect the loss of endogenous hormonal protection in women with DM, as well as later recognition and suboptimal management of cardiovascular conditions in this group, contributing to premature death ^(20)^.

Furthermore, T2DM emerged as a significant modifier in the relationship between age and cause of death. This is in line with findings from the UK Biobank cohort, which demonstrated that individuals with T2DM exhibited signs of accelerated biological aging and had higher all-cause mortality rates ^(21)^. Chronic hyperglycemia, persistent low-grade inflammation, and the accumulation of advanced glycation end products may drive this process and influence the observed mortality patterns ^(21)^. However, not all studies have confirmed such associations. For example, a population-based study conducted in southern Wisconsin, found no significant relationship between T2DM, age at death, and cause-specific mortality after adjusting for confounders such as hypertension, smoking, and dyslipidemia ^(22)^. These results suggest that in some settings, the risk profile of individuals with DM is heavily influenced by coexisting conditions, making it difficult to isolate the direct impact of DM on mortality ^(22)^.

The lack of statistical significance found for other adjustment variables suggests a context-dependent or minimal influence on age-specific mortality patterns in this cohort. Another study observed that socioeconomic status, for instance, impacted DM outcomes through its effect on healthcare access, education, and lifestyle behaviors ^(23)^. Ethnic disparities may also have a role on age-specific mortality patterns; in the United States, African-Americans and Hispanic individuals experience higher rates of DM complications but may differ in their mortality profiles due to both genetic and structural determinants ^(24)^. Furthermore, epigenetic changes induced by chronic stress, poor diet quality, and environmental exposures may modify individuals’ vulnerability to DM complications across different causes of death ^(25)^. The non-significant findings may also reflect sample size limitations or residual confounders, reinforcing the need for larger, stratified studies to unravel the complex interaction between DM, age, and mortality.

One of the key strengths of this study lies in its robust epidemiological design, which integrates a comprehensive population-based approach with an extensive 40-year follow-up dataset. The inclusion of patients from both public and private healthcare settings enhances the representativeness of the sample, ensuring a more accurate reflection of real-world DM-related mortality trends. Furthermore, the meticulous data collection process, rigorously standardized, reinforces the reliability of clinical and demographic records.

Despite its strengths, this study is subject to some limitations. For instance, the lack of clinical confounders (e.g., individuals’ comorbidities) may have impacted the explored relationships. Furthermore, the potential misclassification or reporting bias from death certificates should be mentioned by the nature of data collection. As previously demonstrated in another study conducted by our group, data underreporting DM as the cause of death may lead to biases and interpose confounding factors in this analysis ^(26,27)^.

It should be mentioned that, as 40.6% of eligible individuals were excluded due to missing or inconsistent death records, there is a potential for selection bias that warrants careful consideration. Although exclusions were derived from administrative and logistical issues, the excluded individuals may differ systematically from those included in key sociodemographic or clinical characteristics. Unfortunately, due to the absence of complete identifiers and death records, a direct comparison between included and excluded subjects was not feasible. Nonetheless, this limitation may impact the generalizability of the findings, particularly if the missing cases had distinct mortality patterns or disease severity. Differences in the distribution of causes of death compared with our previous publication reflect a deliberate reclassification based on underlying etiological mechanisms rather than terminal events listed on death certificates. This approach allowed a more coherent and robust analysis of age-related mortality patterns.

In conclusion, this study provides insights into the impact of cause-specific and all-cause mortality on the age at death among patients with DM. Our findings highlight that sex and DM type significantly influenced the age at death, with men and individuals with T2DM showing the most pronounced associations. Patients who died from infectious diseases tended to have a higher age at death, whereas those who died from gastrointestinal diseases tended to present a lower age at death.

These results underscore the necessity of considering both demographic and clinical factors when evaluating mortality patterns in patients with DM. Understanding these associations may help refine risk stratification strategies and improve clinical management, particularly in populations with a high burden of DM-related complications. Future research should explore potential underlying mechanisms and the role of additional factors, such as healthcare access and socioeconomic disparities, in shaping these mortality trends.

## Data Availability

the original data underlying the analyses presented in this study are confidential and are securely maintained by the research team. Due to ethical and privacy considerations, the data are not publicly available. Data collection was approved by an Ethics Committee, as previously described in the manuscript.

## Supplementary table 1

**Supplementary table 1:** Patient’s average death by evaluated categories

Variable	Number of observations/Average and Confidence interval	Average patient’s death age	p-value for variable’s association with patient’s death age	p-value for variable’s association with patient’s death cause
Total	812 (100%)	72.35 ± 14.06	-	
Sex			< 0.01	< 0.01
Men	370 (45.56%)	69.42 ± 18.93	-	
Women	442 (54.44%)	75.44 ± 17.15	-	
Self-reported ethnicity			0.61	0.15
Whites	717 (88.30%)	73.18 ± 13.88	-	
Non-whites	95 (11.70%)	71.25 ± 15.764	-	
Marital status			0.90	0.57
Single	74 (9.11%)	71.92 ± 17.55	-	
Married	585 (72.05%)	73.06 ± 14.19	-	
Widowed	126 (15.52%)	72.14 ± 8.24	-	
Divorced	27 (3.32%)	73.93 ± 11.24	-	
Diabetes type			< 0.01	< 0.01
T1DM	40 (4.93%)	70.22 ± 15.70	-	
T2DM	772 (95.07%)	72.98 ± 10.87	-	
Diabetes duration			0.19	0.10
< 10 years	125 (15.39%)	73.40 ± 12.19	-	
10-19 years	294 (36.21%)	72.16 ± 18.81	-	
20-29 years	278 (34.23%)	72.44 ± 15.29	-	
30-39 years	91 (11.21%)	71.19 ± 14.27	-	
≥ 40 years	24 (2.96%)	71.98 ± 13.18	-	
Diabetes treatment			0.52	0.17
Only diet	40 (4.92%)	74.00 ± 18.27	-	
Only Insulin	412 (50.74%)	73.06 ± 13.17	-	
Only Antidiabetic oral drugs	206 (25.38%)	72.17 ± 15.18	-	
Insulin and Antidiabetic oral drugs	154 (18.96%)	72.49 ± 9.76	-	
BMI			0.13	0.21
Underweight	15 ( 1.84%)	75.92 ± 11.28	-	
Normal	210 (25.88%)	72.17 ± 14.29	-	
Overweight	297 (36.58%)	72.88 ± 15.14	-	
Obesity I	193 (23.77%)	71.88 ± 13.27	-	
Obesity II	59 (7.26%)	71.85 ± 15.19	-	
Obesity III	38 (4.67%)	70.49 ± 15.67	-	
Decade of death			0.43	0.27
1981-1989	34 (4.19%)	71.19 ± 15.29	-	
1990-1999	169 (20.83%)	72.29 ± 14.06	-	
2000-2010	340 (41.84%)	72.54 ± 13.98	-	
2011-2021	269 (33.14%)	71.10 ± 14.87	-	
Death causes			-	-
CVD	167 (20.56%)	72.52 ± 15.24	-	
AC	138 (16.99%)	72.49 ± 18.25	-	
E	45 (5.54%)	69.20 ± 18.13	-	
I	89 (10.96%)	74.22 ± 12.60	-	
N	93 (11.45%)	73.44 ± 15.73	-	
ID	173 (21.30%)	72.27 ± 14.82	-	
GD	107 (13.17%)	70.98 ± 12.24	-	
Numeric variables				
DM duration	14.29 ± 20.78	-	0.09	0.16
BMI	23.98 ± 15.74	-	0.25	0.12
